# Characteristics of gut microbiota structure and composition in diabetic patients from the Chaoshan region of China

**DOI:** 10.1099/jmm.0.002156

**Published:** 2026-07-14

**Authors:** Kun Lin, Baolong Chen, Xinyi Zhang, Ming Fan, Panpan Wang, Yike Wang, Xianhui Cheng, Zeming Ma, Xiaojing Chen, Xingying Xue, Wenting Jia, Songjian Yuan, Bangzhou Zhang, Xiaoman Du, Wenxin Hong, Zhenqiang Hong, Chuanxing Xiao

**Affiliations:** 1 Department of Endocrinology and Metabolism, The First Afﬁliated Hospital of Shantou University Medical College, Shantou 515041, PR China; 2 Xiamen Treatgut Biotechnology Co., Ltd, Xiamen 361101, PR China; 3 Shenyang City University, Shenyang 110100, PR China; 4 School of Pharmacy, Fujian University of Traditional Chinese Medicine, Fuzhou 350122, PR China; 5 Jiangxi Treatgut Biotechnology Co., Ltd, Yichun 336299, PR China; 6 School of Traditional Chinese Medicine, Fujian University of Traditional Chinese Medicine, Fuzhou 350122, PR China; 7 Department of Rehabilitation, Zhongshan Hospital of Xiamen University, Xiamen 361101, PR China

**Keywords:** 16S rDNA, differential diagnosis, gut microbiota, type 1 diabetes mellitus (T1DM), type 2 diabetes mellitus (T2DM)

## Abstract

**Introduction.** Diabetes mellitus, a condition characterized by chronic hyperglycaemia, is categorized into type 1 diabetes mellitus (T1DM) and type 2 diabetes mellitus (T2DM). Recent research has identified a significant association between diabetes and modifications in the gut microbiota.

**Gap Statement.** Although numerous analyses have been conducted on the gut microbiota of patients with diabetes, regional variations due to factors such as lifestyle and dietary habits remain poorly understood.

**Aim.** This study aimed to compare the gut microbiota structures of adult patients with T1DM, T2DM and healthy controls (HCs) in the Chaoshan region of China, providing theoretical support for gut microbiota-based targeted therapies for diabetes in the Chaoshan region.

**Methodology.** This study enrolled 33 patients with T1DM, 35 patients with T2DM and 30 HCs in the Chaoshan region of China. Faecal samples were collected and subjected to 16S rDNA sequencing and bioinformatics analysis.

**Results.** Our data demonstrated significant variations in gut microbiota diversity among individuals with T1DM, T2DM and HC, accompanied by changes in microbial composition across multiple taxonomic levels. Furthermore, linear discriminant analysis effect size analysis identified distinct dominant species within each group: 27 bacterial genera, including *Megasphaera*, were significantly enriched in T2DM patients; 27 bacterial genera, including *Bacteroides*, were significantly enriched in T1DM patients; and 18 bacterial genera, including *Alloprevotella*, were significantly enriched in the HC group. Metabolic pathway analyses using Kyoto Encyclopedia of Genes and Genomes (KEGG) and Clusters of Orthologous Genes (COG) databases demonstrated a significant enrichment of pathways and enzymes associated with starch and sucrose metabolism in both T1DM and T2DM cohorts, compared to HC. Additionally, a diagnostic model based on gut microbiota data at the class level yielded an area under the curve value of 0.861, indicating its high diagnostic efficacy in distinguishing between T1DM and T2DM. Furthermore, an analysis of the abundance of various bacterial phenotypes and probiotic species revealed notable differences among the three groups.

**Conclusion.** This study demonstrates distinct gut microbiota composition, structure and functional profiles in Chaoshan T1DM, T2DM and HC populations, supporting microbiota modulation as a promising therapeutic strategy for diabetes.

## Data Summary

All sequencing data generated in this study (*N*=98) are available under NCBI BioProject accession number PRJNA1313772, with individual accession numbers SAMN50907732 to SAMN50907829 (corresponding numerical IDs: 50907732 to 50907829).

## Introduction

The prevalence of diabetes is increasing annually, due to changes in lifestyle and dietary practices. The International Diabetes Federation reported that in 2021, ~537 million adults globally were affected by diabetes, with projections indicating an increase to 643 million by 2030 and 783 million by 2045 [1]. Notably, China has the highest number of individuals with diabetes worldwide, with the prevalence rate surging from below 1% in 1980 to 12.4% in 2018 [2]. Diabetes mellitus is a chronic condition characterized by persistent hyperglycaemia and is categorized into type 1 diabetes mellitus (T1DM) and type 2 diabetes mellitus (T2DM). While T1DM accounts for ~5% of the overall diabetes population, the absolute number of individuals affected remains substantial. T1DM predominantly manifests in children and young adults, although it can also occur in adulthood [3,4]. Although many cases of diabetes are effectively managed with existing treatments, a subset of patients still experiences inadequate glycaemic control and new complications [5,6]. Approximately 30%–40% of individuals with diabetes develop diabetic nephropathy, with one-third of these patients ultimately advancing to end-stage renal disease, which often requires dialysis or kidney transplantation [7,8].

The gut microbiota is involved in numerous physiological processes, including metabolism and immune function, and plays a critical role in regulating the intestinal mucosal barrier and intestinal permeability [9]. Alterations in intestinal permeability have been linked to the pathogenesis of T1DM [10]. A substantial body of research has indicated that individuals with diabetes experience gut microbiota dysbiosis. Notably, children with T1DM demonstrated decreased microbial diversity and a diminished presence of bacteria responsible for the production of butyrate and lactate [11]. Adult patients diagnosed with T1DM exhibited alterations in gut microbiota, which correlated with glycaemic control indices [12]. Furthermore, individuals with T2DM also displayed gut microbiota dysbiosis [13], which resulted in heightened intestinal permeability and chronic inflammation, thereby aggravating insulin resistance [14]. Early postnatal administration of probiotics may reduce the risk of islet autoimmunity in children [15]. Probiotics and synbiotics have served as complementary therapies for diabetes management [16]. Interventions targeted at diabetes have demonstrated effective glycaemic control in patients with T2DM, along with a favourable safety profile [17,18]. Therefore, T1DM and T2DM can be managed through intervention strategies aimed at restoring gut microbiome balance, enhancing intestinal barrier function and reducing inflammation. However, it is important to note that the gut microbiota changes in T1DM and T2DM exhibit distinct patterns [19], and the composition of probiotic strains varies widely. Consequently, probiotic intervention strategies for T1DM and T2DM should be tailored to address these differences, necessitating further analysis of the distinct gut microbiota compositions associated with these two conditions.

Although numerous analyses have focused on the gut microbiota of patients with diabetes, diabetes incidence exhibits regional variations influenced by factors like lifestyle and dietary habits [20]. In this study, we used 16S rDNA sequencing and bioinformatics analysis to compare the gut microbiota structures of adult patients with T1DM, T2DM and healthy controls (HCs) in the Chaoshan region of China. Our findings provide theoretical support for gut microbiota-based targeted therapies for diabetes in this region.

## Methods

### Study cohorts

This study was approved by the Ethical Committee of the First Affiliated Hospital of Shantou University Medical College (approval number: B-2021-225). All participants provided written informed consent. Between January 2023 and January 2024, a total of 33 patients with T1DM and 35 with T2DM were enrolled from the Department of Endocrinology and Metabolism at the First Affiliated Hospital of Shantou University Medical College, China. Additionally, 30 HCs were recruited as the normal control group. They were matched for age and gender with the patient groups and were undergoing health examinations during the same period. Demographic and clinical data were collected, including age, age at onset, gender, body mass index (BMI), smoking and alcohol history, dietary and family history, diabetes-related complications and comorbidities, diabetes treatment regimens and medication usage.

### Inclusion and exclusion criteria

Inclusion criteria for HC: (1) Age range: 18 to 65 years, inclusive (regardless of gender). (2) Health status: No significant medical conditions, as confirmed by comprehensive physical examination, including but not limited to cardiovascular, hepatic, renal, malignant or autoimmune diseases. (3) Glycaemic control: Normal fasting plasma glucose and glycated haemoglobin (HbA1c) levels. (4) BMI: 18.5 to 24 kg m^−^². (5) Informed consent: Voluntary participation with a signed informed consent form.

Exclusion criteria for HC: (1) Established or suspected diabetes mellitus, as evidenced by medical history, physical examination or laboratory findings. (2) Pregnancy or lactation. (3) Severe cardiac, hepatic or renal systems. (4) Mental illness that may compromise the subject’s suitability for the study or the integrity of the data. (5) Concurrent enrolment in other clinical trials. (6) History of heavy smoking, alcohol abuse or significant lifestyle changes within the past 6 months. (7) Gastrointestinal infections, chronic inflammatory bowel diseases, coeliac disease, active malignancies (particularly gastrointestinal cancers) or congenital/acquired immunodeficiencies. (8) Use of antibiotics or probiotics within 1 month prior to enrolment.

Inclusion criteria for T1DM: (1) Aged between 18 and 65 years, regardless of gender. (2) Newly diagnosed and untreated, meeting the World Health Organization criteria for T1DM: symptomatic with random blood glucose (RBG) ≥11.1 mmol l^−1^ (≥200 mg dl^−1^), fasting blood glucose (FBG) ≥7.0 mmol l^−1^ (≥126 mg dl^−1^), 2 h post-75 g oral glucose tolerance test blood glucose ≥11.1 mmol l^−1^ (≥200 mg dl^−1^) or HbA1c≥6.5% (48 mmol mol^−1^).

Exclusion criteria for T1DM: (1) Type 2 diabetes or other specific types of diabetes. (2) Pregnant or lactating women. (3) Severe cardiac, hepatic or renal dysfunction. (4) Gastrointestinal infections, chronic inflammatory bowel diseases (including Crohn’s disease and ulcerative colitis), coeliac disease, active malignancies (particularly gastrointestinal cancers) and both congenital and acquired immunodeficiencies. (5) Mental illness that may impair the patient’s ability to comply with the study or the accuracy of the data. (6) Concurrent participation in other clinical trials. (7) Extensive history of smoking, alcohol abuse or significant lifestyle changes within the past 6 months. (8) Use of antibiotics, probiotics or similar medications within 1 month prior to enrolment.

Inclusion criteria for T2DM: (1) Aged 18 to 65 years, regardless of gender. (2) Newly diagnosed with T2DM who have not been treated with oral hypoglycaemic agents or insulin (according to the 2024 American Diabetes Association diagnostic criteria: HbA1c≥6.5%, FBG≥7.0 mmol l^−1^, 2 h blood glucose≥11.1 mmol l^−1^ or patients with classic symptoms accompanied by RBG≥11.1 mmol l^−1^).

Exclusion criteria for T2DM: (1) Type 1 diabetes or other specific types of diabetes. (2) Pregnant or lactating women. (3) Severe cardiac, hepatic or renal impairment. (4) Gastrointestinal infections, chronic inflammatory bowel diseases, coeliac disease, active malignancies (particularly gastrointestinal cancers) or congenital/acquired immunodeficiencies. (5) Mental disorders that may compromise the patient’s ability to comply with the study procedures or the accuracy of the data. (6) Concurrent participation in other clinical trials. (7) History of long-term smoking, alcohol abuse or major lifestyle changes within the past 6 months. (8) Use of antibiotics, probiotics or other intestinal flora modifiers within 1 month prior to enrolment.

### Samples

Blood samples: Prior to enrolment, fasting and postprandial blood samples were collected from the participants for the assessment of various parameters, including glucose metabolism (HbA1c, blood glucose and C-peptide), insulin resistance index (HOMA-IR), fasting insulin levels, liver and kidney function and blood lipids (total cholesterol, triglycerides, LDL-C and HDL-C). Additionally, faecal samples: Faecal samples were collected from the participants, ensuring avoidance of contamination from urine and dust. Uncontaminated portions were selected and stored at room temperature in a preservation solution (Xiamen Treatgut Biotechnology Co., Ltd., China) for 16S rDNA gene sequencing.

### Nucleic acid extraction

DNA was extracted from faecal samples using the QIAamp DNA Stool Extraction Mini Kit (Qiagen, Germany). Subsequently, the extracted DNA was subjected to quality control via agarose gel electrophoresis, followed by quantification using a UV spectrophotometer.

### High-throughput sequencing

PCR amplification of the V3-V4 region of the 16S rRNA gene was performed using the primer pair 341F and 806R. The forward primer sequence was 5′-GTGCCAGCMGCCGCGGTAA-3′, and the reverse primer sequence was 5′-GGACTACNVGGGTWTCTAAT-3′. The PCR amplification protocol involved an initial denaturation step at 95 °C for 2 min, followed by a total of 25 cycles, with each cycle consisting of 95 °C for 30 s, annealing at 55 °C for 30 s, extension at 72 °C for 30 s and a final extension at 72 °C for 10 min [21]. PCR products were quantified and pooled using the Quant-iT PicoGreen dsDNA Assay Kit on the Promega QuantiFluor fluorometer. Dual-end sequencing was conducted on the Illumina sequencing platform (250PE) following standard procedures. All sequencing data generated in this study (*N*=98) are available under NCBI BioProject accession number PRJNA1313772, with individual accession numbers SAMN50907732 to SAMN50907829 (corresponding numerical IDs: 50907732 to 50907829).

### Bioinformatics analysis

High-quality, optimized sequences were clustered into operational taxonomic units (OTUs) using USEARCH software, with a sequence similarity threshold set at 97%. An OTU abundance table was then generated based on these clusters. For bacterial annotation analysis, the representative sequences of the OTUs were aligned to the silva database (version 138.1, accessible at https://www.arb-silva.de/documentation/release-1381/). Next, alpha diversity was calculated using QIIME2 based on the OTUs, and beta diversity was determined based on Bray–Curtis distances between samples. Correlation analysis of the identified differential bacterial genera was conducted using the rcorr function from the R package Hmisc. Results with correlation coefficients exceeding 0.4 were selected for visualization, which was subsequently performed using Cytoscape software.

### Construction of a predictive model

A predictive classifier to distinguish between T1DM, T2DM and HC was developed by leveraging differences in gut microbiota. Using the ‘RandomForest’ package in R, we trained the model on genera that had a relative abundance greater than 0.1%. To ensure the model’s generalization capability, the combined dataset from the T1DM, T2DM and HC groups was randomly split into training and test sets in a 7 : 3 ratio. A fivefold cross-validation approach was performed on the training set. Additionally, the ‘Boruta’ package in R was used to identify significant bacterial genera for classification. The final model was built using these selected features. The area under the ROC (receiver operating characteristic) curve (AUC) was calculated to evaluate the model’s discriminative performance.

### Statistical analysis

Descriptive results were presented as mean±sd. Comparisons between two groups for bacterial taxon abundances were performed using the non-parametric Mann–Whitney U test. The non-parametric Kruskal–Wallis H test was employed to determine if there were significant differences in *α*-diversity indices between groups. Permutational multivariate analysis of variance was utilized to assess statistical differences in *β*-diversity among groups. Using 16S rDNA gene sequencing data and the Kyoto Encyclopedia of Genes and Genomes (KEGG) and Clusters of Orthologous Genes (COG) databases, we applied PICRUSt to predict the functional potential of the gut microbiota. Given that simultaneous statistical testing across hundreds of microbial taxa and functional pathways significantly increases the risk of false discoveries, we rigorously applied the Benjamini–Hochberg (BH) procedure to control the false discovery rate (FDR) and ensure the robustness of our results. A *P* value of <0.05 was considered statistically significant.

## Results

### Baseline characteristics of the study cohorts

The study cohort comprised 33 T1DM patients (20F/13M; median age: 24.8 years; disease duration: 0.6 years; BMI: 25.36), 35 T2DM patients (19F/16M; median age: 39.18 years; disease duration: 1.8 years; BMI: 26.97) and 30 HCs (12F/18M; median age: 36.2 years; BMI: 21.54). Detailed baseline characteristics are summarized in Table 1.

**Table 1.** Clinical characteristics of the study cohorts

Clinical characteristics		HC (*n*=30)	T1DM (*n*=33)	T2DM (*n*=35)
Gender	Female	12	20	19
	Male	18	13	16
Age (years)	Median	32.6	24.8	39.18
	Range	26.7–45.1	19.4–29.7	29.46–44.14
Disease course (years)	Median	–	0.6	1.8
	Range	–	0.3–1.1	0.8–2.6
BMI	Median	21.54	25.36	26.97
	Range	19.86–23.14	19.37–30.78	21.48–28.99

### Differences in gut microbiota between patients with T1DM and T2DM and HC in the Chaoshan region of China

**Fig. 1.**
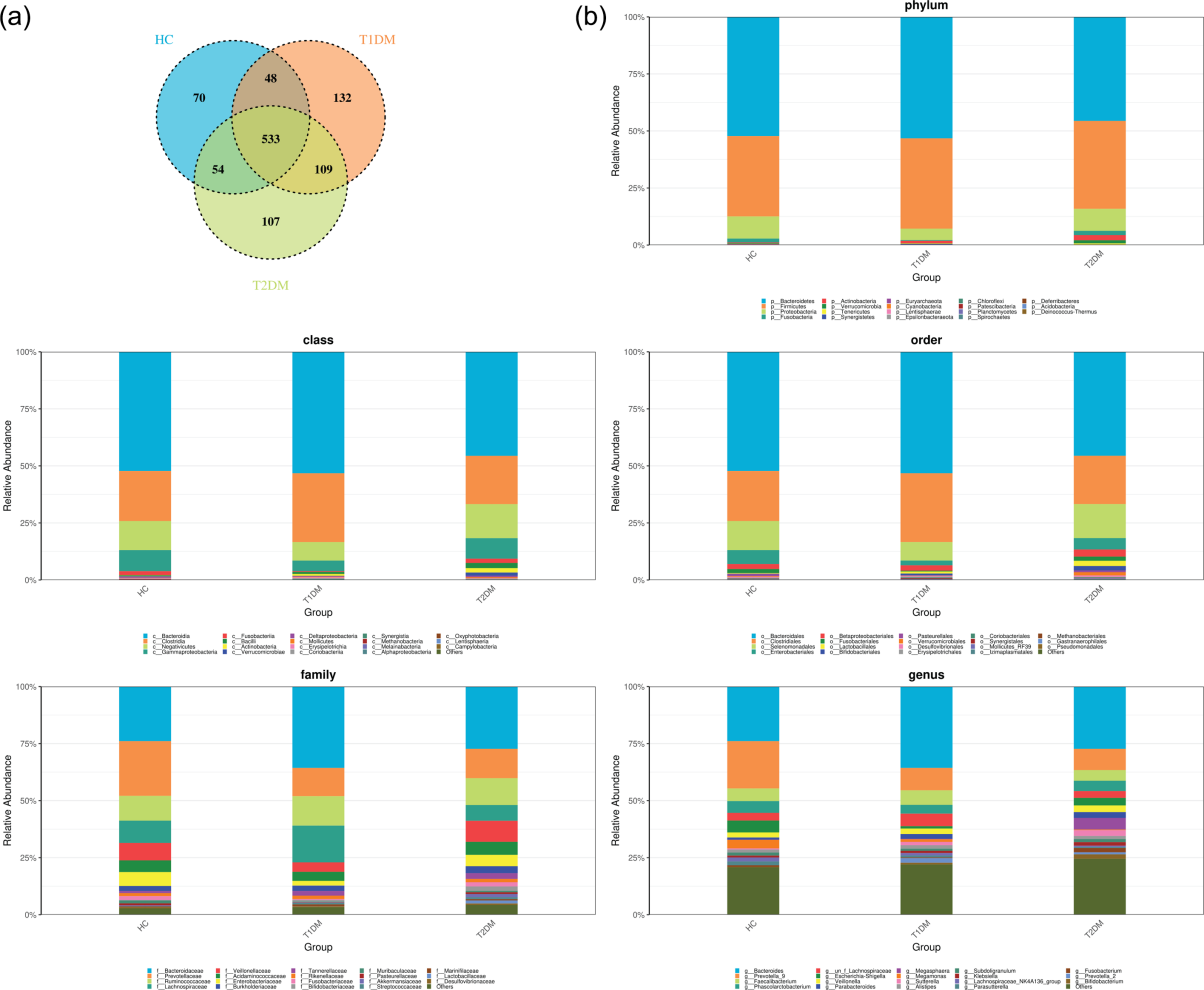
Analysis of gut microbiota in patients with T1DM and T2DM compared to HC. (**a**) Venn diagram depicting OTU distribution of gut microbiota in T1DM, T2DM patients and HC. (**b**) Multi-level bacterial taxa distribution histogram comparing gut microbiota in T1DM, T2DM patients and HC. T1DM, type 1 diabetes mellitus; T2DM, type 2 diabetes mellitus; HC, healthy control; OTU, operational taxonomic unit.

To investigate differences in gut microbiota composition between patients with T1DM or T2DM and HC in the Chaoshan region of China, we performed 16S rDNA sequencing on faecal samples. A Venn diagram showed 533 shared OTUs among the three groups, with 270 unique OTUs in the HC group, 132 in the T1DM group and 107 in the T2DM group (Fig. 1a). Significant differences were observed in the bacterial composition at the phylum, class, order, family and genus levels among the three groups. At the family level, *Bacteroidaceae* was significantly more abundant in both T1DM and T2DM patients, while *Prevotellaceae* was significantly reduced. The relative abundance of *Lachnospiraceae* was increased in T1DM patients but significantly decreased in T2DM patients. At the genus level, *Bacteroides* was significantly elevated in both T1DM and T2DM patients, whereas *Prevotella_9* was significantly reduced (Fig. 1b).

Additionally, comparative analysis (T1DM vs. HC, T2DM vs. HC and T1DM vs. T2DM) further confirmed significant disparities in bacterial composition (Fig. S1, available in the online Supplementary Material). Microbial community analysis identified 581 core OTUs shared between T1DM patients and HC, with 124 HC-specific and 241 T1DM-specific OTUs. Comparisons between T2DM patients and HC groups revealed 587 core OTUs (118 HC-specific and 216 T2DM-specific OTUs), and the T1DM vs. T2DM comparison yielded 642 core OTUs (180 T1DM-specific and 161 T2DM-specific OTUs). In addition to variations in OTU numbers, we observed significant differences in microbial composition at the genus level among all groups. Notably, T1DM patients exhibited a higher abundance of *Bacteroides* but a lower abundance of *Megasphaera* compared to T2DM patients.

### Gut microbiota diversity between patients with T1DM or T2DM and HC in the Chaoshan region of China

**Fig. 2.**
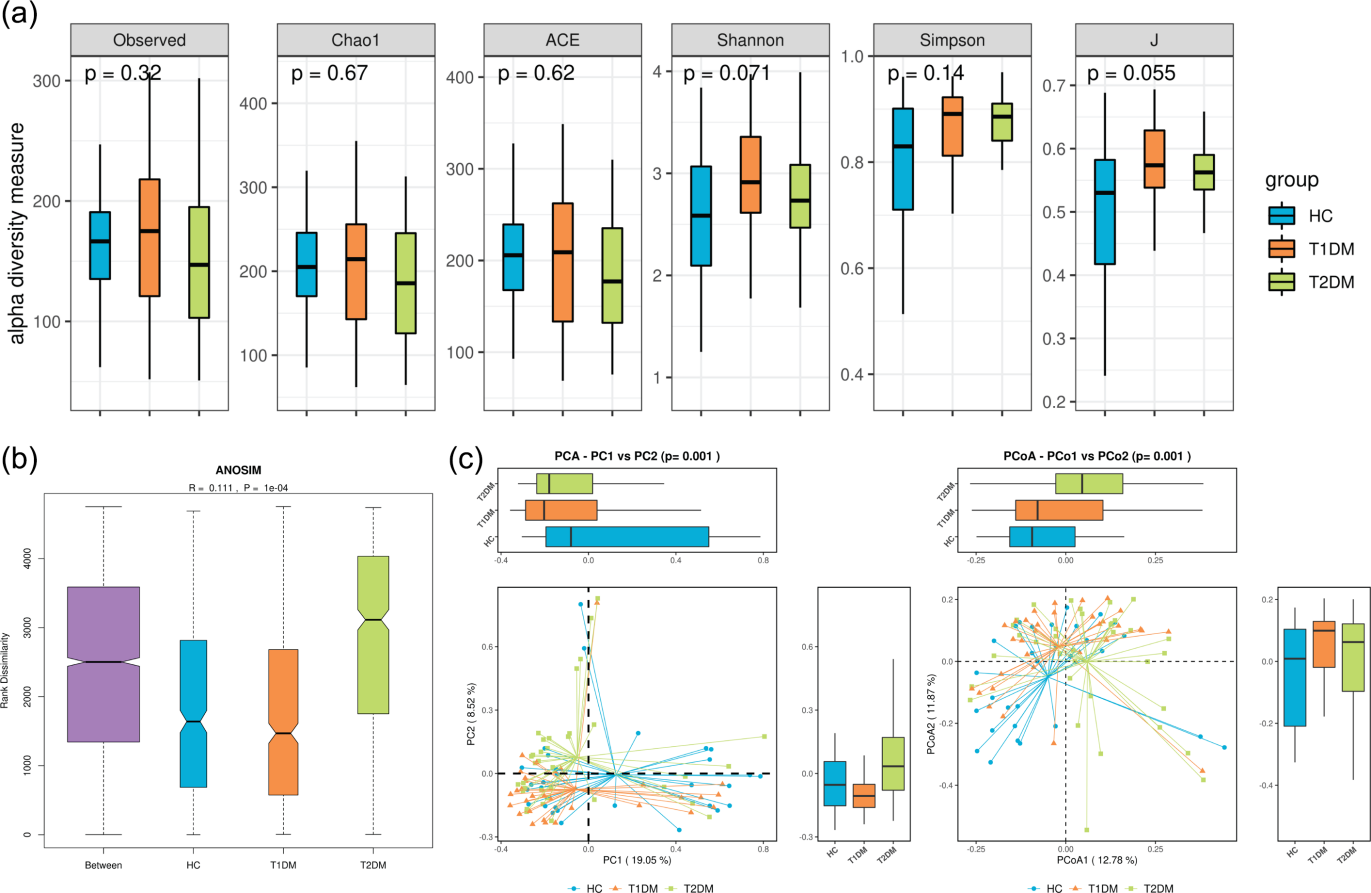
Gut microbiota diversity in patients with T1DM and T2DM versus HC. (**a**) Alpha diversity revealing differences in gut microbiota between T1DM, T2DM patients and HC. (**b**) ANOSIM comparing gut microbiota in T1DM, T2DM patients and HC. (**c**) Beta diversity uncovering heterogeneity of gut microbiota in T1DM, T2DM patients and HC. T1DM, type 1 diabetes mellitus; T2DM, type 2 diabetes mellitus; HC, healthy control.

The analysis of *α*-diversity, as assessed by the Shannon, Simpson and J indices, revealed that there were no statistically significant differences among the three groups (Fig. 2a). Analysis of similarity (ANOSIM) revealed a significant difference in similarity among the three groups (R=0.111, *P*=1e-04) (Fig. 2b). Principal component analysis and principal coordinates analysis, based on bacterial community composition, indicated significant differences in *β*-diversity (Fig. 2c). Further analysis confirmed significant differences in *α*-diversity between all pairs of groups (T1DM vs. HC, T2DM vs. HC and T1DM vs. T2DM). Additionally, significant differences were observed in both the ANOSIM index and *β*-diversity (Fig. S2).

### Patients with T1DM and T2DM exhibited distinct bacterial taxa in their gut microbiota compared to HC

**Fig. 3.**
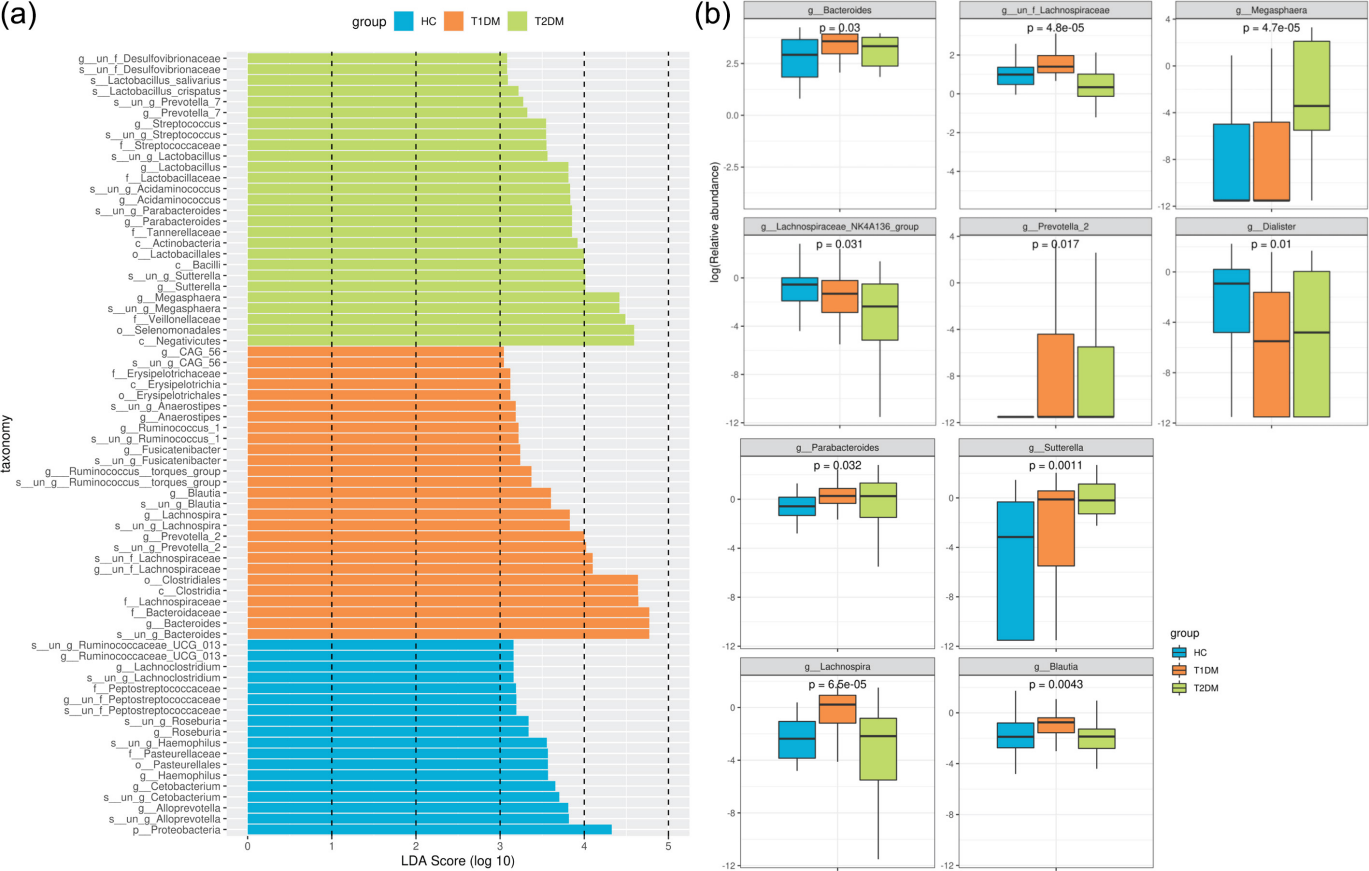
LEfSe analysis of gut microbiota in patients with T1DM and T2DM compared to HC. (**a**) Enrichment analysis of differential bacterial genera of T1DM, T2DM patients and HC. (**b**) Top 10 different bacterial genera of T1DM, T2DM patients and HC. LEfSe, linear discriminant analysis effect size; T1DM, type 1 diabetes mellitus; T2DM, type 2 diabetes mellitus; HC, healthy control.

The *β*-diversity analysis confirmed differences in gut microbiota among patients with T1DM, T2DM and the HC. Subsequent LEfSe analysis (linear discriminant analysis score threshold >3) identified specific bacterial taxa contributing to these differences. The results revealed that 27 genera, including *Megasphaera* and *Sutterella*, were enriched in T2DM patients, while 27 genera, such as *Prevotella_2* and *Bacteroides*, were enriched in T1DM patients. In contrast, 18 genera, including *Cetobacterium* and *Alloprevotella*, were enriched in HC (Fig. 3a). A taxonomic cladogram demonstrated significant differences in gut microbiota abundance from the phylum to genus level across all three groups (Fig. S3A).

Further analysis identified the top 10 genera with significant differences in relative abundance. Compared to HC, the relative abundance of genera such as *Bacteroides*, *Prevotella_2*, *Parabacteroides* and *Sutterella* was significantly higher in both T1DM and T2DM patients. Conversely, genera such as *Lachnospiraceae_NK4A136_group* and *Dialister* were significantly lower in these patient groups. *Megasphaera* abundance was significantly higher in T2DM patients but unchanged in T1DM patients (Fig. 3b). Additionally, *Actinomyces* abundance was significantly higher in T1DM patients but remained unchanged in T2DM patients (Fig. S3B). Significant differences in bacterial abundance were observed at the phylum, class, order and family levels across all groups (Fig. S4). Additionally, LEfSe analysis identified the top 10 genera with significant differences between T1DM and HC and T2DM and HC, as well as T1DM and T2DM (Fig. S5).

### Patients with T1DM and T2DM demonstrated distinct gut microbiota metabolic pathways compared to HC

**Fig. 4.**
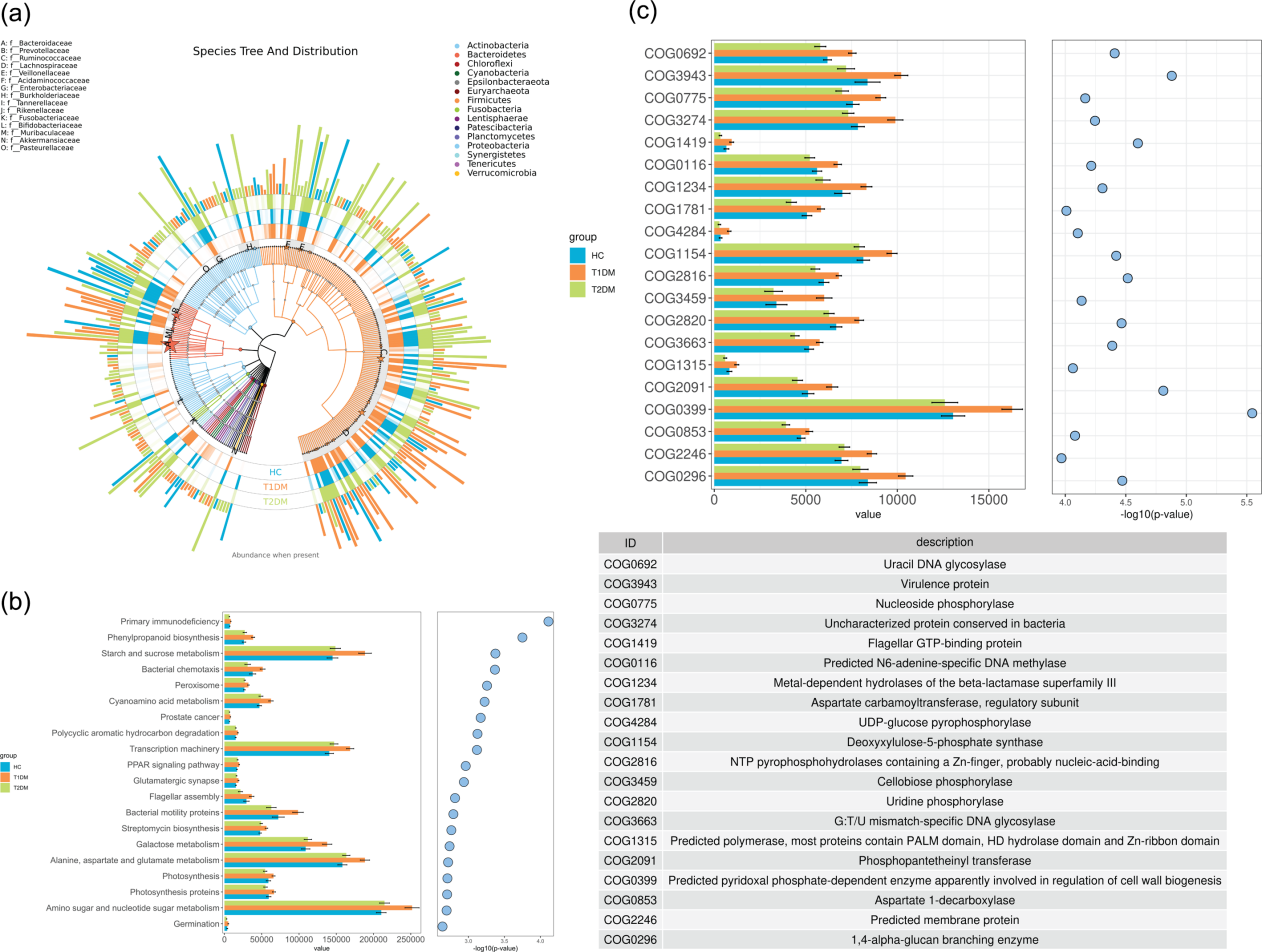
Metabolic pathways in gut microbiota of patients with T1DM and T2DM compared to HC. (**a**) Overview of gut microbiota composition across all samples. (**b, c**) Differential metabolic pathways in gut microbiota of T1DM, T2DM patients and HC based on KEGG and COG analysis. T1DM, type 1 diabetes mellitus; T2DM, type 2 diabetes mellitus; HC, healthy control; KEGG, Kyoto Encyclopedia of Genes and Genomes; COG, Clusters of Orthologous Genes.

Next, we visualized the gut microbiota composition and distribution across different groups using a circular cladogram, which depicted phylogenetic relationships among bacterial taxa and their distribution across samples (Fig. 4a). Based on 16S rDNA sequencing data, we predicted and compared metabolic pathways using the KEGG database to explore functional variations in the gut microbiota among different groups. The results indicated that, compared to HC, multiple metabolic pathways were significantly enriched in both T1DM and T2DM groups, with the T1DM group demonstrating greater enrichment than the T2DM group, particularly in pathways such as starch and sucrose metabolism and amino sugar and nucleotide sugar metabolism (Fig. 4b). Further analysis using the COG database for functional categorization focused on the top 20 most abundant COG categories. The findings revealed that, compared to HC, the abundance of genes categorized under specific COG functions, such as UDP-glucose pyrophosphorylase (COG4284) and 1,4-alpha-glucan branching enzyme (COG0296), was significantly higher in the T1DM group and significantly lower in the T2DM group (Fig. 4c). Additionally, both KEGG pathway and COG functional category analyses identified numerous features that differed significantly across all pairwise comparisons (T1DM vs. HC, T2DM vs. HC and T1DM vs. T2DM) (Fig. S6).

### Gut microbiota at various taxonomic levels demonstrated differing diagnostic efficacy in distinguishing patients with T1DM, T2DM and HC

**Fig. 5.**
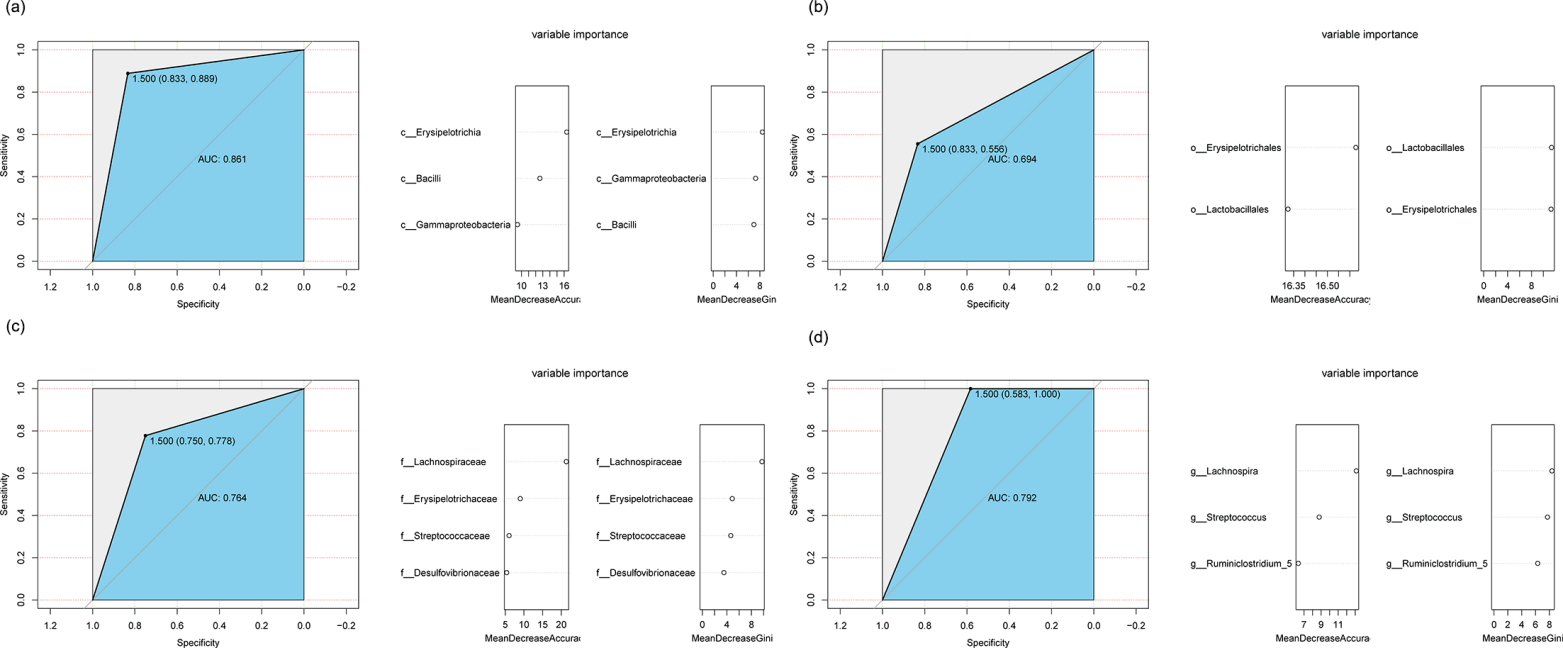
Discriminative models for T1DM and T2DM patients based on gut microbiota. (**a–d**) Discriminative performance of models constructed using differential bacterial taxa at the class, order, family and genus levels for T1DM and T2DM patients. T1DM, type 1 diabetes mellitus; T2DM, type 2 diabetes mellitus.

We established diagnostic models based on characteristic genera to differentiate between T1DM, T2DM and HC. For differentiating between T2DM and T1DM, the diagnostic efficacy of the models, as assessed by the AUC values, was 0.861 at the class level, 0.694 at the order level, 0.764 at the family level and 0.792 at the genus level. Notably, the highest diagnostic efficacy was observed at the class level (Fig. 5). Additionally, we constructed a diagnostic model for distinguishing HC from T1DM. The ROC curve analysis revealed that the highest diagnostic efficacy was achieved at the class level, with an AUC value of 0.798 (Fig. S7). For differentiating between HC and T2DM patients, the ROC curve analysis indicated that the highest diagnostic efficacy was at the genus level, with an AUC value of 0.750 (Fig. S8).

### Variations in aerobic and biofilm-forming bacteria in T1DM, T2DM and HC

**Fig. 6.**
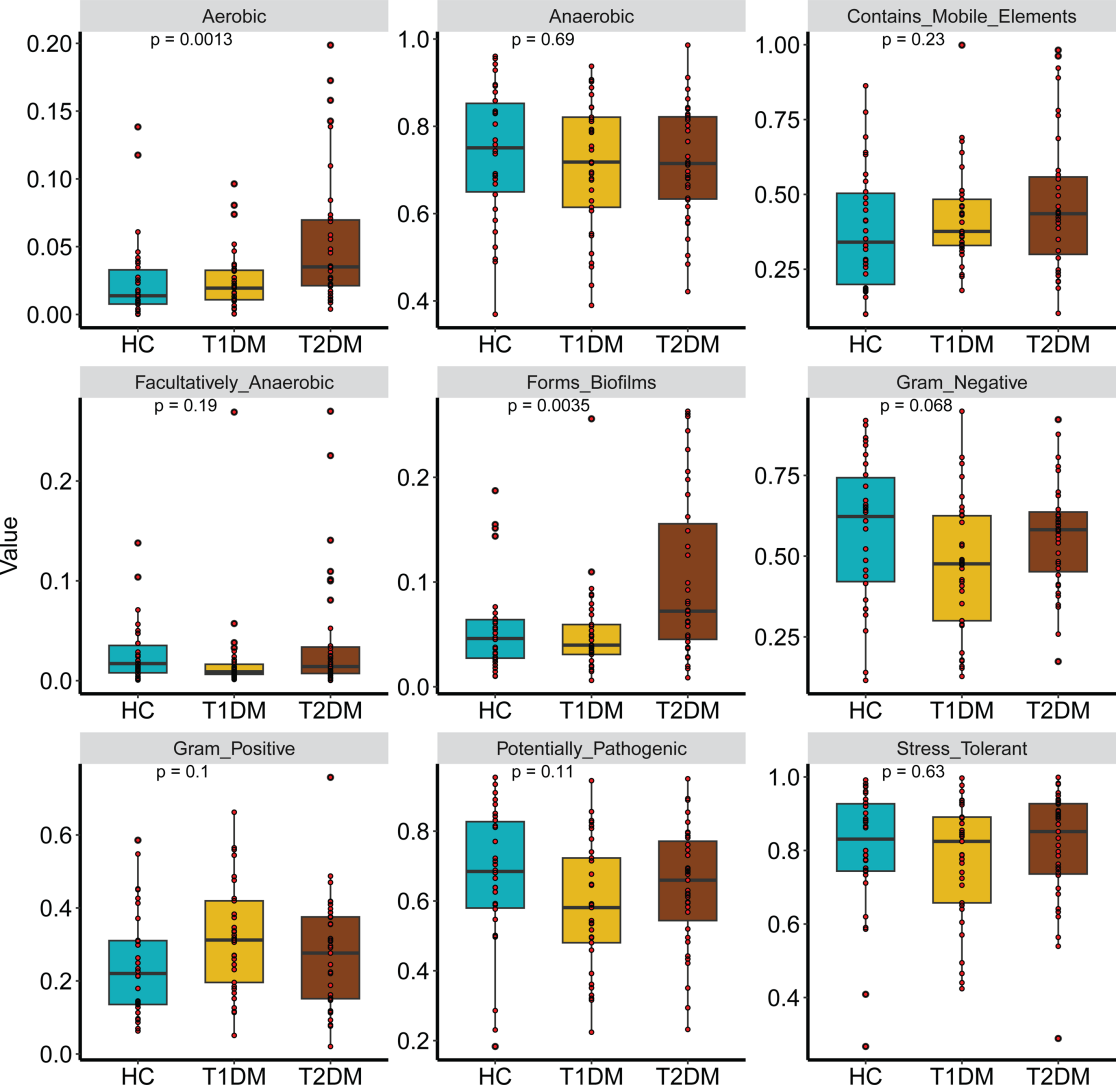
Comparative analysis of bacterial genus abundance across gut microbiota phenotypes in patients with T1DM, T2DM and HC.

This study further analysed the differences in bacterial genus across different gut microbiota phenotypes among patients with T1DM, T2DM and HC in the Chaoshan region of China. The results indicated that the relative abundance of aerobic bacteria was significantly increased in both T1DM and T2DM patients. Bacteria that produce biofilms showed a significant increase in abundance in the T2DM group but a significant decrease in the T1DM group (Fig. 6). In addition, the relative abundance of bacteria containing mobile genetic elements and Gram-positive bacteria exhibited an increasing trend in both T2DM and T1DM patients. The relative abundance of anaerobic bacteria, Gram-negative bacteria and potentially pathogenic bacteria demonstrated a decreasing trend in both T1DM and T2DM patients.

### Gut probiotic abundance observed between T1DM, T2DM patients and HC in the Chaoshan region of China

**Fig. 7.**
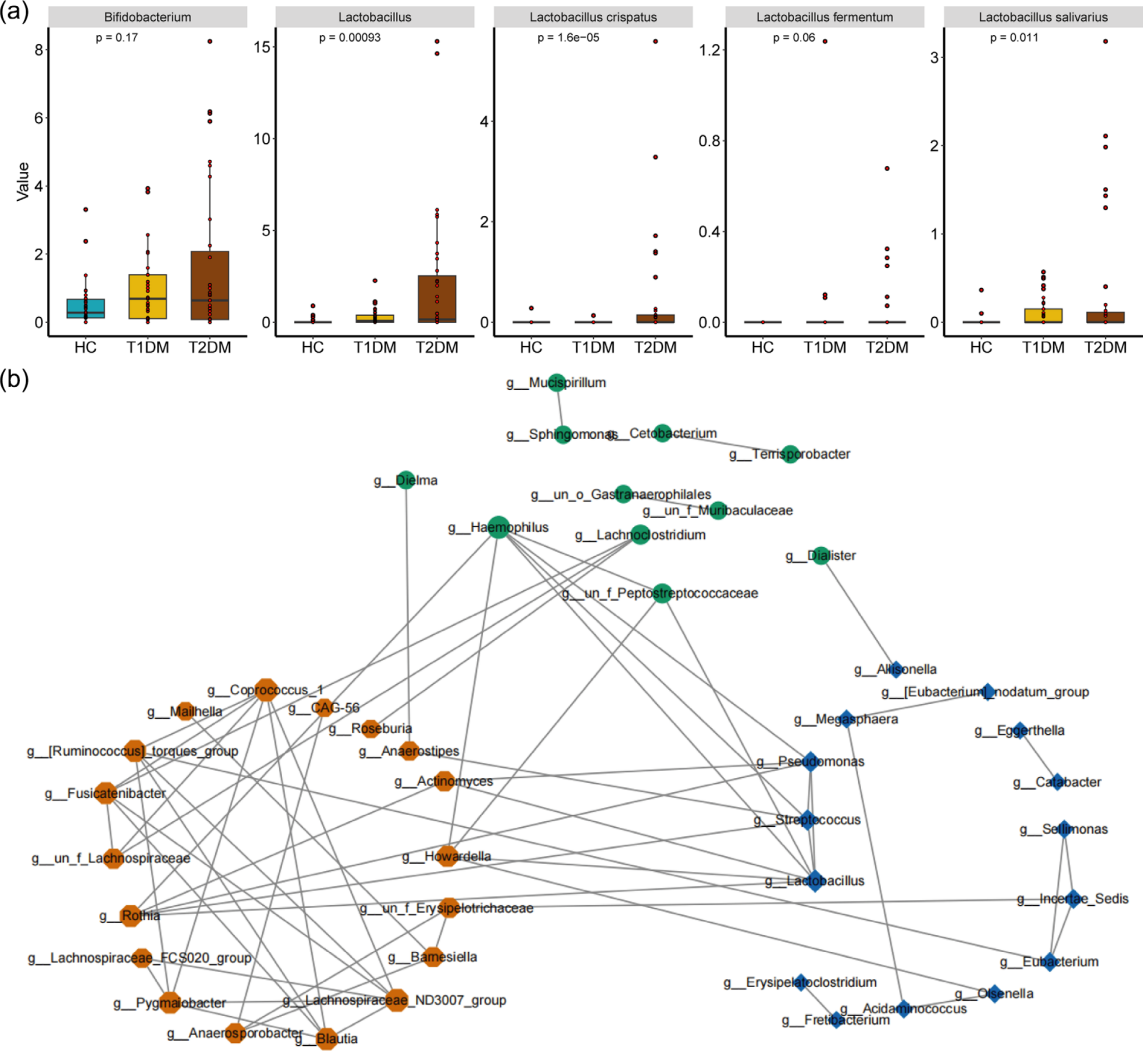
Analysis of intestinal probiotic abundance in patients with T1DM, T2DM and HC. (**a**) Box plot showing differences in intestinal probiotic abundance between patients with T1DM, T2DM and HC. (**b**) Correlation analysis of intestinal probiotic abundance in patients with T1DM, T2DM and HC. T1DM, type 1 diabetes mellitus; T2DM, type 2 diabetes mellitus; HC, healthy control.

Analysis of five beneficial bacterial taxa revealed that *Bifidobacterium* was most abundant in T2DM patients and least abundant in HC. *Lactobacillus* and *Lactobacillus crispatus* abundances were both significantly higher in T2DM patients compared to both the T1DM and HC groups. *Lactobacillus salivarius* was most abundant in T1DM patients, showing statistically significant differences from the other two groups (Fig. 7a). Correlation analysis indicated that in HC, the genera *Lachnoclostridium* and *Haemophilus* were strongly associated with T1DM and T2DM, respectively, suggesting that they could play a role in distinguishing diabetes types (Fig. 7b). These findings suggested that the above-mentioned genera could represent biomarkers for distinguishing between the two types of diabetes.

## Discussion

Our study utilized 16S rDNA sequencing to compare the gut microbiota of patients with T1DM and T2DM. Significant differences were observed in gut microbiota diversity and composition among individuals with T1DM, T2DM and HC. Specifically, *Megasphaera* and *Sutterella* were predominantly enriched in T2DM patients, while *Prevotella_2* and *Bacteroides* were enriched in T1DM patients. Conversely, *Cetobacterium* and *Alloprevotella* were enriched in the HC group. Furthermore, compared to HC, the relative abundance of genera such as *Bacteroides*, *Prevotella_2*, *Parabacteroides* and *Sutterella* was significantly higher in both T1DM and T2DM patients. In contrast, the relative abundance of genera *Lachnospiraceae_NK4A136_group* and *Dialister* was significantly lower in these patient groups. Notably, compared to HC, the abundance of the genus *Megasphaera* was significantly higher in T2DM patients but remained unchanged in T1DM patients. Additionally, multiple metabolic pathways were significantly enriched in both the T1DM and T2DM groups compared to HC, with the T1DM group demonstrating higher enrichment than the T2DM group, particularly in pathways such as starch and sucrose metabolism and amino sugar and nucleotide sugar metabolism. Diagnostic models that utilized characteristic genera to differentiate among T1DM, T2DM and HC exhibited AUC values of 0.792, 0.798 and 0.750, respectively, indicating moderate diagnostic accuracy.

A meta-analysis has revealed significant differences in the gut microbiota structure and diversity between patients with T2DM and HC, with gut microbiota demonstrating high predictability for diabetes [22]. Similarly, numerous studies have identified variations in the gut microbiome of patients with T1DM compared to HC [11]. Gut microbiota dysbiosis and a relative decrease in *α*-diversity have been associated with T1DM [23]. Additionally, a study comparing the gut microbiota composition of adult patients with T1DM and T2DM has also shown differences between the two conditions [19]. Collectively, these findings suggested that both T1DM and T2DM induced alterations in the gut microbiota, albeit in distinct manners, implying that the intervention strategies targeting the gut microbiota may differ for the two types of diabetes. Gut microbiota dysbiosis in diabetic patients is associated with multiple factors, including the use of antidiabetic medication [24], hypertension [25], lipid profiles [26] and impaired liver function [27,28]. Modulation of the gut microbiome exerted a statistically significant effect on diastolic blood pressure in individuals with T2DM [29]. Hence, targeted modulation of the gut microbiota in the early stages of the disease may mitigate the progression of diabetes-related complications [26,30, 31].

In this study, the diagnostic discriminative models we developed, based on differential bacterial taxa identified at the class, order, family and genus levels, demonstrated notable efficacy in accurately distinguishing between T1DM and T2DM, T1DM and HC and T2DM and HC. Significantly, the class-level model exhibited the highest diagnostic efficacy for differentiating T1DM from T2DM, achieving an AUC of 0.861. This highlighted the substantial potential of class-level microbial markers in improving diabetes subtype diagnostic precision. These identified bacterial genera represented robust microbial biomarkers for the early and precise identification of diabetes and its subtypes. This capability was particularly critical given diabetes heterogeneity and the inherent challenges of achieving accurate early-stage diagnosis. Utilizing these biomarkers could enable clinicians to stratify patients more effectively, tailor treatment strategies and monitor disease progression with enhanced accuracy. Furthermore, these microbial markers constituted promising targets for gut microbiota-targeted interventions. Given the mounting evidence linking gut microbiota dysbiosis to diabetes pathogenesis, modulating the microbiota through dietary interventions, probiotics, prebiotics or faecal microbiota transplantation represented a potential novel therapeutic strategy [32]. Our study provided a foundation for future research into the therapeutic applications of these microbial markers, ultimately aiming to develop personalized treatments targeting the underlying microbial imbalances associated with diabetes.

Previous studies have demonstrated that *Bacteroides* exhibited high abundance in both diabetic mouse models [33] and paediatric patients with T1DM [34,35]. *Bacteroides* plays a pivotal role in the onset and progression of T1DM, potentially through the production of glutamic acid decarboxylase and possibly inducing glutamic acid decarboxylase autoimmunity via molecular mimicry [36]. Kovatcheva-Datchary *et al*. [37] found that individuals with a higher abundance of *Prevotella copri* exhibited improved glucose metabolism in response to diet-induced hyperglycaemia. Therefore, the abundance of *Prevotella* may contribute to the regulation of glucose metabolism in diabetes. Our findings revealed a higher abundance of the bacterial genera *Bacteroides* and *Prevotella_2* in patients with T1DM and T2DM compared to healthy individuals. We propose that this may underlie the greater enrichment of starch and sucrose metabolism and amino sugar and nucleotide sugar metabolism observed in T1DM and T2DM patients. *Megasphaera*, a dominant bacterial genus in patients with T2DM [38], is known to enhance intestinal lipid absorption and promote high-fat diet consumption [39]. A *Megasphaera* species, *Megasphaera elsdenii*, induced colonic immune dysregulation by promoting dendritic cell-mediated Th1 and Th17 polarization via the TLR4/NF-κB/IRF4 pathway [40]. This inflammatory shift aligned with the elevated Th1/Th2 and Th17/Treg ratios observed in patients with T2DM compared to HCs [41,42]. *Sutterella*, which has been found to be more abundant in patients with T2DM and T1DM compared with HC, demonstrated beneficial effects on glycometabolism [43]. However, strains of *Sutterella* exhibited mild proinflammatory activity but did not compromise the integrity of intestinal epithelial cell monolayers in coculture experiments [44]. In addition, we examined the relative abundance of *Actinomyces* in each sample and found that it was consistently high in the majority of samples from T1DM patients, whereas it only reached high levels in a minority of samples from the HC and T2DM groups. *Actinomyces* demonstrated significant protective effects against autoimmune hepatitis and primary sclerosing cholangitis [45]. Furthermore, Ma *et al*. conducted high-throughput sequencing of faecal samples from T1DM patients and observed a notable increase in the abundance of *Actinobacteria* in T1DM rats. Notably, the presence or increased abundance of *Actinobacteria* was associated with inflammatory responses, posing potential harm to the host [46]. Conversely, *Cetobacterium* was enriched in the HC group. Previous work has suggested that the production of acetate by *Cetobacterium* contributed to the improvement of glucose homeostasis and elevated insulin expression through parasympathetic activation [47]. We hypothesized that supplementing diabetic patients with *Cetobacterium* may effectively ameliorate their insulin secretion deficiency. *Cetobacterium*, as a potential probiotic, presents promising applications in diabetes management and warrants comprehensive investigation to assess the efficacy and safety of its supplementation in enhancing diabetes outcomes.

KEGG analysis revealed significant enrichment of the primary immunodeficiency pathway in T1DM patients. As an autoimmune disease, T1DM may involve impaired immune regulatory function induced by intestinal microbiota. This dysregulation could lead to the breakdown of immune tolerance. For example, it may cause abnormal recognition of self-antigens, potentially promoting autoantibody production and exacerbating pancreatic *β*-cell damage [48]. Concurrently, the starch and sucrose metabolism pathway demonstrated differential enrichment across all three groups. Gut microbiota ferment undigested dietary carbohydrates via this pathway, generating short-chain fatty acids (SCFAs) that enhance insulin sensitivity through GPR43/41 receptor activation [49]. Furthermore, we observed abnormalities in the PPAR signalling pathway within the T2DM cohort. Given that PPARs (peroxisome proliferator-activated receptors) serve as master regulators of lipid homeostasis and insulin sensitivity [50], their impaired activation by microbial metabolites (e.g. SCFAs and bile acid derivatives) [51] may contribute to ectopic lipid accumulation and insulin resistance – core pathophysiological drivers of T2DM progression.

This study has certain limitations. First, the cross-sectional design employed in our study imposed a key limitation. As this approach captures data at a single timepoint, we cannot determine whether gut microbiota alterations contribute to diabetes development/progression or whether diabetes drives microbiota changes. Moreover, this design precludes longitudinal assessment of gut microbiota evolution in diabetic patients. Given diabetes’ chronic progressive nature and dynamic metabolic shifts during disease progression, understanding temporal microbiota dynamics in relation to metabolic changes is essential for elucidating disease mechanisms. Future longitudinal studies are needed to assess how temporal microbiota shifts correlate with metabolic trajectories or therapeutic responses in diabetic patients. Secondly, while non-parametric approaches (Kruskal–Wallis test with BH-FDR correction) appropriately accommodate small-sample distributions, the constrained cohort size (notably in the T1DM subgroup, *n*=33) fundamentally limits statistical sensitivity. We therefore consider these findings as preliminary biological evidence necessitating external validation in expanded cohorts. Furthermore, T1DM was more prevalent among younger individuals, whereas T2DM was more common in older adults; age is a known factor influencing gut microbiota composition [52]. Future research should be conducted with a larger sample size and include a more comprehensive analysis, considering various factors such as age, BMI, physiological conditions, diet and lifestyle habits, as well as their impacts on gut microbiota.

Through a comparative analysis of the gut microbiota in cohorts of patients with T2DM, T1DM and HC from the Chaoshan region of China, this study identified significant differences in the gut microbiota profiles between T2DM and T1DM patients and HC. These differences may be associated with the functional and metabolic pathways of the gut microbiota and exhibit certain diagnostic and discriminatory efficacy for T1DM and T2DM. Our findings highlighted the potential for therapeutic interventions aimed at modulating the gut microbiota and its metabolites to improve outcomes for T2DM and T1DM, offering valuable insights for targeted intervention strategies.

## Supplementary material

10.1099/jmm.0.002156 Supplementary Material.

## Abbreviations

BH: Benjamini–Hochberg
BMI: body mass index
COG: Clusters of Orthologous Genes
FBG: fasting blood glucose
FDR: false discovery rate
HCs: healthy controls
KEGG: Kyoto Encyclopedia of Genes and Genomes
OTUs: operational taxonomic units
PPARs: peroxisome proliferator-activated receptors
RBG: random blood glucose
ROC: receiver operating characteristic
SCFAs: short-chain fatty acids
T1DM: type 1 diabetes mellitus
T2DM: type 2 diabetes mellitus
