# Supplementary material for: Characteristics of gut microbiota structure and composition in diabetic patients from the Chaoshan region of China
Source: J Med Microbiol. 2026 Jul 14;75(7):002156. doi: 10.1099/jmm.0.002156 (PMC13367372; doi:10.1099/jmm.0.002156)
Supplement: Supplementary Material. [file jmm-75-02156-s001.pdf]

Supplementary figures

Figure S1. Differential genera analysis in patients with T1DM versus HC, T2DM versus HC, and T1DM versus T2DM. (A) Venn diagram (left panel) depicting the OTU distribution of gut microbiota in T1DM patients compared to HC. Bar chart (right panel) illustrating the bacterial distribution at the genus level between T1DM patients and HC. (B) Venn diagram (left panel) displaying OTU distribution in gut microbiota of T2DM patients relative to HC. Bar chart (right panel) representing the bacterial distribution at the genus level for T2DM patients versus HC. (C) Venn diagram (left panel) showing OTU distribution in gut microbiota of T1DM patients relative to T2DM patients. Bar chart (right panel) representing the bacterial distribution at the genus level for T1DM patients versus T2DM patients. T1DM, type 1 diabetes mellitus; T2DM, type 2 diabetes mellitus; HC, healthy controls; OTU, operational taxonomic unit.

Figure S2. Diversity analysis of gut microbiota in patients with T1DM versus HC, T2DM versus HC, and T1DM versus T2DM. (A) The α-diversity index, ANOSIM, and β-diversity index of gut microbiota in T1DM patients compared to HC. (B) The α-diversity index, ANOSIM, and β-diversity index of gut microbiota in T2DM patients compared to HC. (C) The α-diversity index, ANOSIM, and β-diversity index of gut microbiota in T1DM patients compared to T2DM patients. T1DM, type 1 diabetes mellitus; T2DM, type 2 diabetes mellitus; HC, healthy controls.

Figure S3. (A) Cladogram of differential bacterial genera in the gut microbiota of patients with T1DM and T2DM compared to HC. (B) Bar chart showing the relative abundance of bacterial genus *Actinomyces* in each sample from the T1DM, T2DM, and HC groups. T1DM, type 1 diabetes mellitus; T2DM, type 2 diabetes mellitus; HC, healthy controls.

Figure S4. Significant differences in bacterial composition at the (A) phylum, (B) class, (C) order, and (D) family levels in the gut microbiota of patients with T1DM, T2DM, and HC. T1DM, type 1 diabetes mellitus; T2DM, type 2 diabetes mellitus; HC, healthy controls.

Figure S5. LEfSe analysis of gut microbiota in patients with T1DM versus HC, T2DM versus HC, and T1DM versus T2DM. (A) Enrichment analysis of differential bacterial

genera in the gut microbiota of T1DM versus HC (left), T2DM versus HC (middle), and T1DM versus T2DM (right). (B) The top 10 most significantly different features at the genus level in the gut microbiota of T1DM versus HC (top), T2DM versus HC (middle), and T1DM versus T2DM (bottom). T1DM, type 1 diabetes mellitus; T2DM, type 2 diabetes mellitus; HC, healthy controls.

Figure S6. Analysis of metabolic pathways in the gut microbiota of patients with T1DM versus HC, T2DM versus HC, and T1DM versus T2DM. (A) Differential metabolic pathways in the gut microbiota of T1DM versus HC (left), T2DM versus HC (middle), and T1DM versus T2DM (right) based on KEGG analysis. (B) Differential metabolic pathways in the gut microbiota of T1DM versus HC (left), T2DM versus HC (middle), and T1DM versus T2DM (right) based on COG analysis. T1DM, type 1 diabetes mellitus; T2DM, type 2 diabetes mellitus; HC, healthy controls.

Figure S7. Discriminative models for distinguishing patients with T1DM from HC based on gut microbiota. (A) Discriminative performance of the model constructed using differential bacteria at the class level. (B) Discriminative performance of the model constructed using differential bacteria at the order level. (C) Discriminative performance of the model constructed using differential bacteria at the family level. (D) Discriminative performance of the model constructed using differential bacteria at the genus level. T1DM, type 1 diabetes mellitus; T2DM, type 2 diabetes mellitus; HC, healthy controls.

Figure S8. Discriminative models for differentiating patients with T2DM from HC based on gut microbiota composition. (A) Model performance utilizing differential bacterial taxa at the class level. (B) Model performance utilizing differential bacterial taxa at the order level. (C) Model performance utilizing differential bacterial taxa at the family level. (D) Model performance utilizing differential bacterial taxa at the genus level. T1DM, type 1 diabetes mellitus; T2DM, type 2 diabetes mellitus; HC, healthy controls.

Figure S1

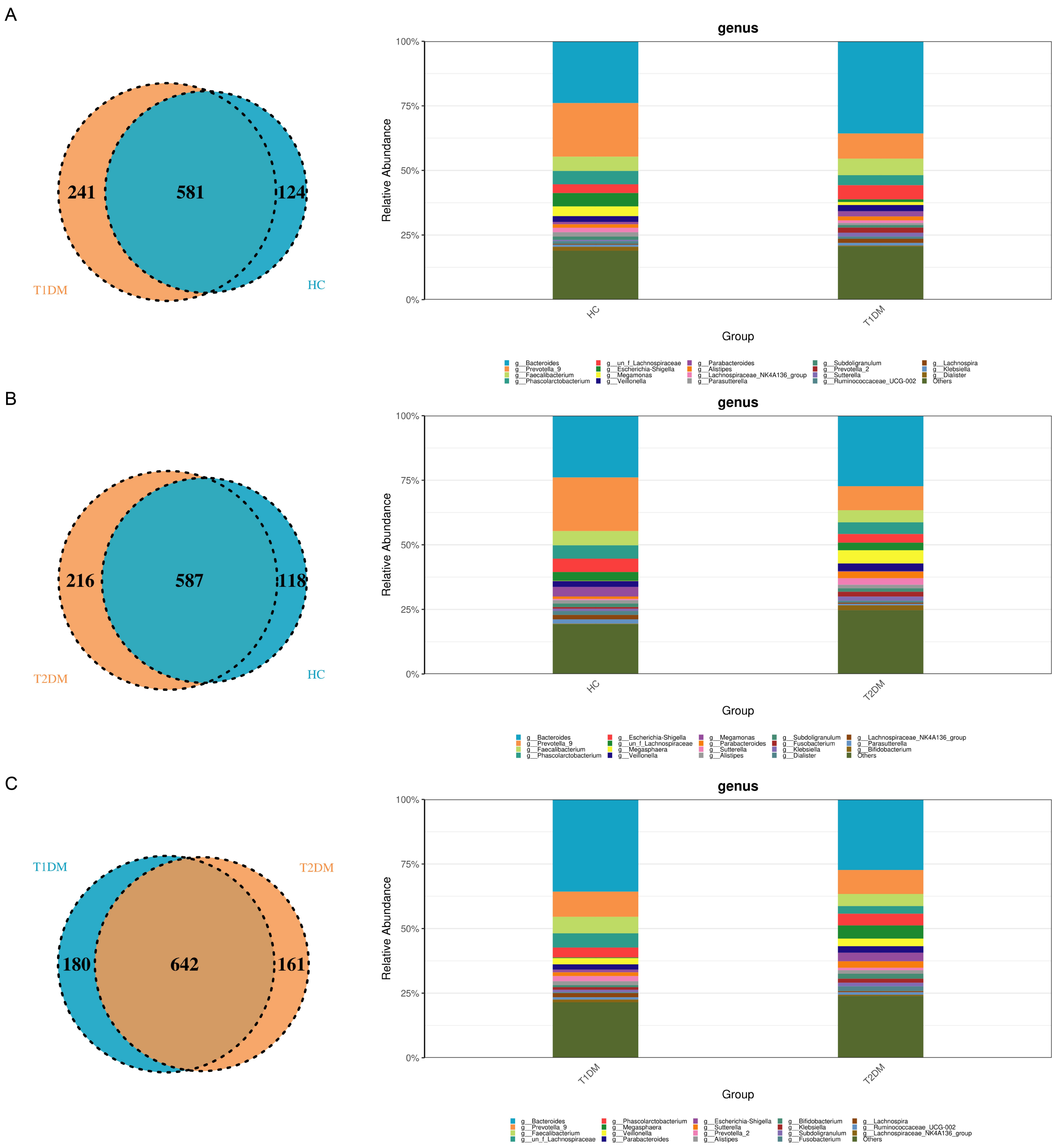

A
241
581
124
T1DM
HC
genus
Relative Abundance
100%
75%
50%
25%
0%
HC
T1DM
Group
g__Bacteroides
g__Prevotella_9
g__Faecalibacterium
g__Phascolarctobacterium
g__un_f_Lachnospiraceae
g__Escherichia-Shigella
g__Megamonas
g__Veillonella
g__Parabacteroides
g__Alistipes
g__Lachnospiraceae_NK4A136_group
g__Parasutterella
g__Subdoligranulum
g__Prevotella_2
g__Sutterella
g__Ruminococcaceae_UCG-002
g__Lachnospira
g__Klebsiella
g__Dialister
Others
B
216
587
118
T2DM
HC
genus
Relative Abundance
100%
75%
50%
25%
0%
HC
T2DM
Group
g__Bacteroides
g__Prevotella_9
g__Faecalibacterium
g__Phascolarctobacterium
g__Escherichia-Shigella
g__un_f_Lachnospiraceae
g__Megasphaera
g__Veillonella
g__Megamonas
g__Parabacteroides
g__Sutterella
g__Alistipes
g__Subdoligranulum
g__Fusobacterium
g__Klebsiella
g__Dialister
g__Lachnospiraceae_NK4A136_group
g__Parasutterella
g__Bifidobacterium
Others
C
T1DM
T2DM
180
642
161
genus
Relative Abundance
100%
75%
50%
25%
0%
T1DM
T2DM
Group
g__Bacteroides
g__Prevotella_9
g__Faecalibacterium
g__un_f_Lachnospiraceae
g__Phascolarctobacterium
g__Megasphaera
g__Veillonella
g__Parabacteroides
g__Escherichia-Shigella
g__Sutterella
g__Prevotella_2
g__Alistipes
g__Bifidobacterium
g__Klebsiella
g__Subdoligranulum
g__Fusobacterium
g__Lachnospira
g__Ruminococcaceae_UCG-002
g__Lachnospiraceae_NK4A136_group
Others

Figure S2

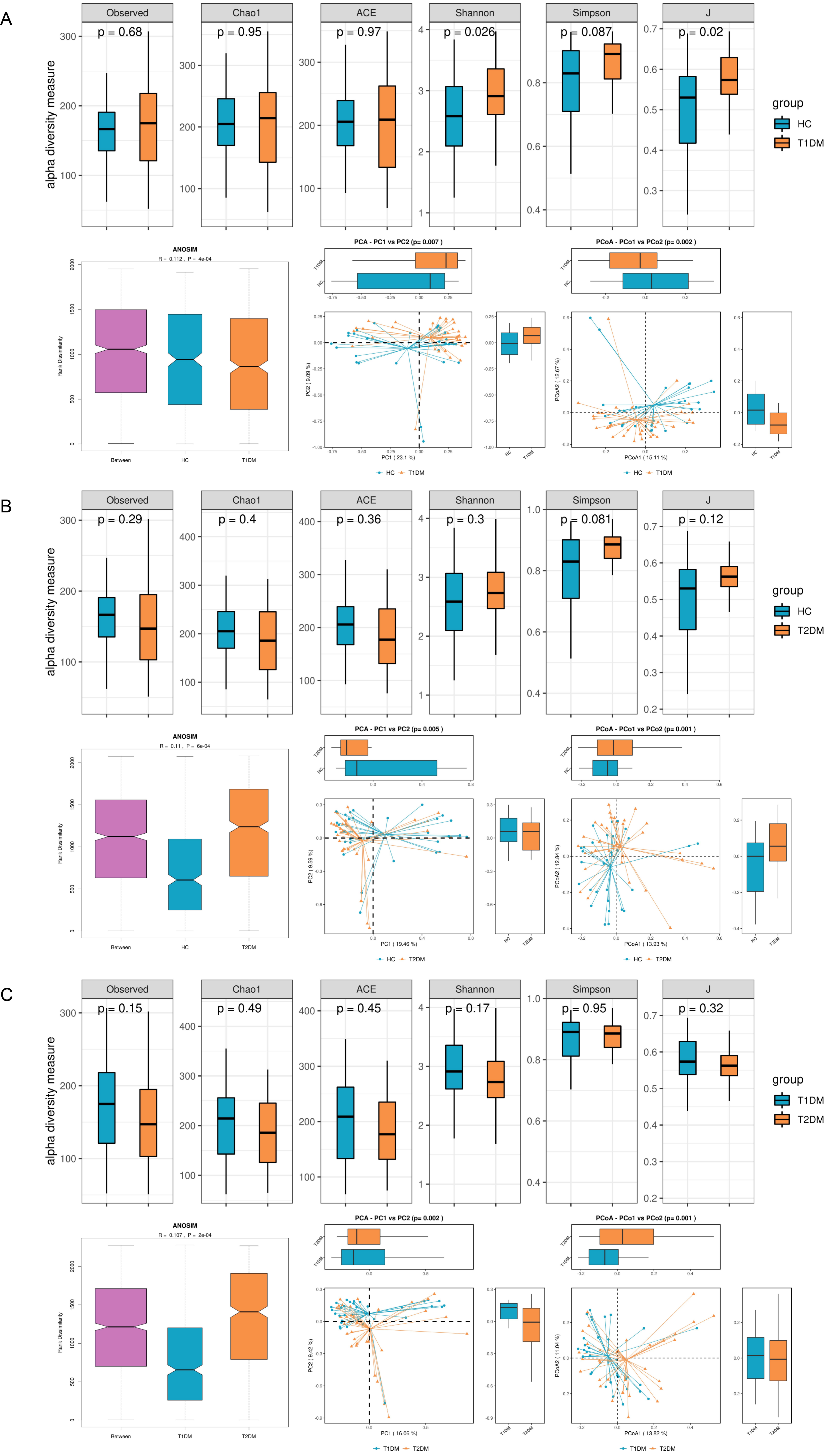
A
Observed
p = 0.68
Chao1
p = 0.95
ACE
p = 0.97
Shannon
p = 0.026
Simpson
p = 0.087
J
p = 0.02
alpha diversity measure
group
HC
T1DM
ANOSIM
R = 0.112 , P = 4e-04
Rank Dissimilarity
Between
HC
T1DM
PCA - PC1 vs PC2 (p= 0.007)
PC1 (23.1 %)
PC2 (9.09 %)
PCoA - PCo1 vs PCo2 (p= 0.002)
PCoA1 (15.11 %)
PCoA2 (12.67 %)
B
Observed
p = 0.29
Chao1
p = 0.4
ACE
p = 0.36
Shannon
p = 0.3
Simpson
p = 0.081
J
p = 0.12
alpha diversity measure
group
HC
T2DM
ANOSIM
R = 0.11 , P = 6e-04
Rank Dissimilarity
Between
HC
T2DM
PCA - PC1 vs PC2 (p= 0.005)
PC1 (19.46 %)
PC2 (9.59 %)
PCoA - PCo1 vs PCo2 (p= 0.001)
PCoA1 (13.93 %)
PCoA2 (12.84 %)
C
Observed
p = 0.15
Chao1
p = 0.49
ACE
p = 0.45
Shannon
p = 0.17
Simpson
p = 0.95
J
p = 0.32
alpha diversity measure
group
T1DM
T2DM
ANOSIM
R = 0.107 , P = 2e-04
Rank Dissimilarity
Between
T1DM
T2DM
PCA - PC1 vs PC2 (p= 0.002)
PC1 (16.06 %)
PC2 (9.42 %)
PCoA - PCo1 vs PCo2 (p= 0.001)
PCoA1 (13.82 %)
PCoA2 (11.04 %)

Figure S3

A

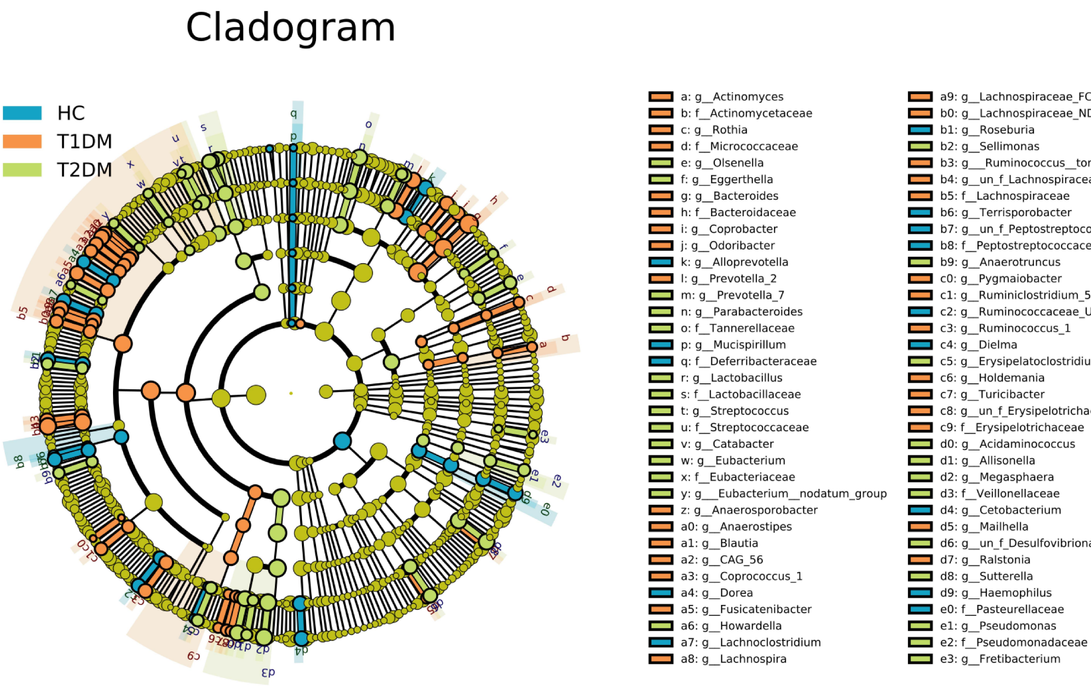

Cladogram
HC
T1DM
T2DM
a: g__Actinomyces
b: f__Actinomycetaceae
c: g__Rothia
d: f__Micrococcaceae
e: g__Olsenella
f: g__Eggerthella
g: g__Bacteroides
h: f__Bacteroidaceae
i: g__Coprobacter
j: g__Odoribacter
k: g__Alloprevotella
l: g__Prevotella_2
m: g__Prevotella_7
n: g__Parabacteroides
o: f__Tannerellaceae
p: g__Mucispirillum
q: f__Deferribacteraceae
r: g__Lactobacillus
s: f__Lactobacillaceae
t: g__Streptococcus
u: f__Streptococcaceae
v: g__Catabacter
w: g__Eubacterium
x: f__Eubacteriaceae
y: g__Eubacterium__nodatum_group
z: g__Anaerosporobacter
a0: g__Anaerostipes
a1: g__Blautia
a2: g__CAG_56
a3: g__Coprococcus_1
a4: g__Dorea
a5: g__Fusicatenibacter
a6: g__Howardella
a7: g__Lachnoclostridium
a8: g__Lachnospira
a9: g__Lachnospiraceae_FC
b0: g__Lachnospiraceae_ND
b1: g__Roseburia
b2: g__Sellimonas
b3: g__Ruminococcus__tor
b4: g__un_f_Lachnospiracea
b5: f__Lachnospiraceae
b6: g__Terrisporobacter
b7: g__un_f_Peptostreptoco
b8: f__Peptostreptococcace
b9: g__Anaerotruncus
c0: g__Pygmaiobacter
c1: g__Ruminiclostridium_5
c2: g__Ruminococcaceae_U
c3: g__Ruminococcus_1
c4: g__Dielma
c5: g__Erysipelatoclostridiu
c6: g__Holdemania
c7: g__Turicibacter
c8: g__un_f_Erysipelotricha
c9: f__Erysipelotrichaceae
d0: g__Acidaminococcus
d1: g__Allisonella
d2: g__Megasphaera
d3: f__Veillonellaceae
d4: g__Cetobacterium
d5: g__Mailhella
d6: g__un_f_Desulfovibriona
d7: g__Ralstonia
d8: g__Sutterella
d9: g__Haemophilus
e0: f__Pasteurellaceae
e1: g__Pseudomonas
e2: f__Pseudomonadaceae
e3: g__Fretibacterium

B

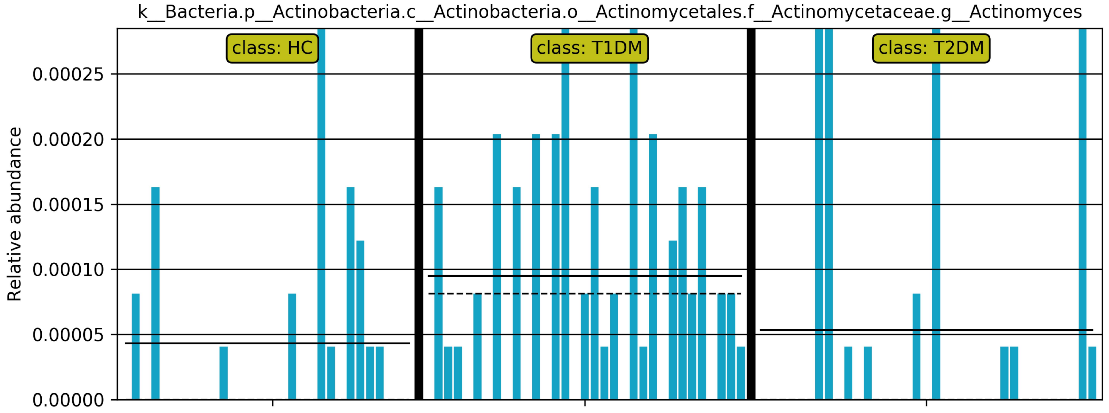

k__Bacteria.p__Actinobacteria.c__Actinobacteria.o__Actinomycetales.f__Actinomycetaceae.g__Actinomyces
class: HC
class: T1DM
class: T2DM
Relative abundance
0.00025
0.00020
0.00015
0.00010
0.00005
0.00000

Figure S4

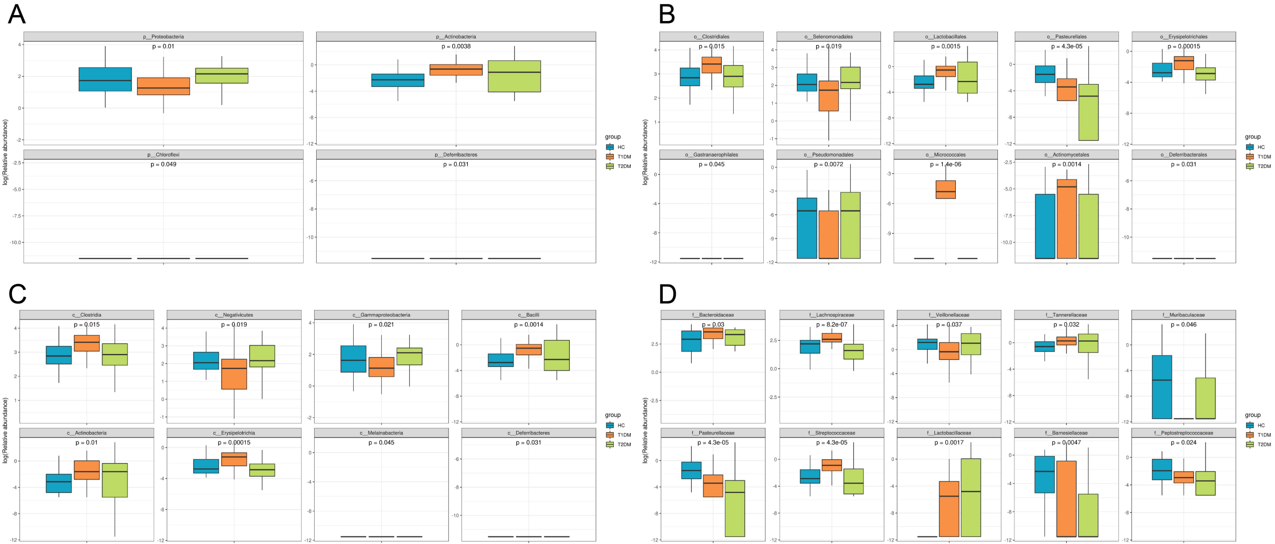
A
p__Proteobacteria
p = 0.01
p__Actinobacteria
p = 0.0038
p__Chloroflexi
p = 0.049
p__Deferribacteres
p = 0.031
log(Relative abundance)
group
HC
T1DM
T2DM
B
o__Clostridiales
p = 0.015
o__Selenomonadales
p = 0.019
o__Lactobacillales
p = 0.0015
o__Pasteurellales
p = 4.3e-05
o__Erysipelotrichales
p = 0.00015
o__Gastranaerophilales
p = 0.045
o__Pseudomonadales
p = 0.0072
o__Micrococcales
p = 1.4e-06
o__Actinomycetales
p = 0.0014
o__Deferribacterales
p = 0.031
C
c__Clostridia
p = 0.015
c__Negativicutes
p = 0.019
c__Gammaproteobacteria
p = 0.021
c__Bacilli
p = 0.0014
c__Actinobacteria
p = 0.01
c__Erysipelotrichia
p = 0.00015
c__Melainabacteria
p = 0.045
c__Deferribacteres
p = 0.031
D
f__Bacteroidaceae
p = 0.03
f__Lachnospiraceae
p = 8.2e-07
f__Veillonellaceae
p = 0.037
f__Tannerellaceae
p = 0.032
f__Muribaculaceae
p = 0.046
f__Pasteurellaceae
p = 4.3e-05
f__Streptococcaceae
p = 4.3e-05
f__Lactobacillaceae
p = 0.0017
f__Barnesiellaceae
p = 0.0047
f__Peptostreptococcaceae
p = 0.024

Figure S5

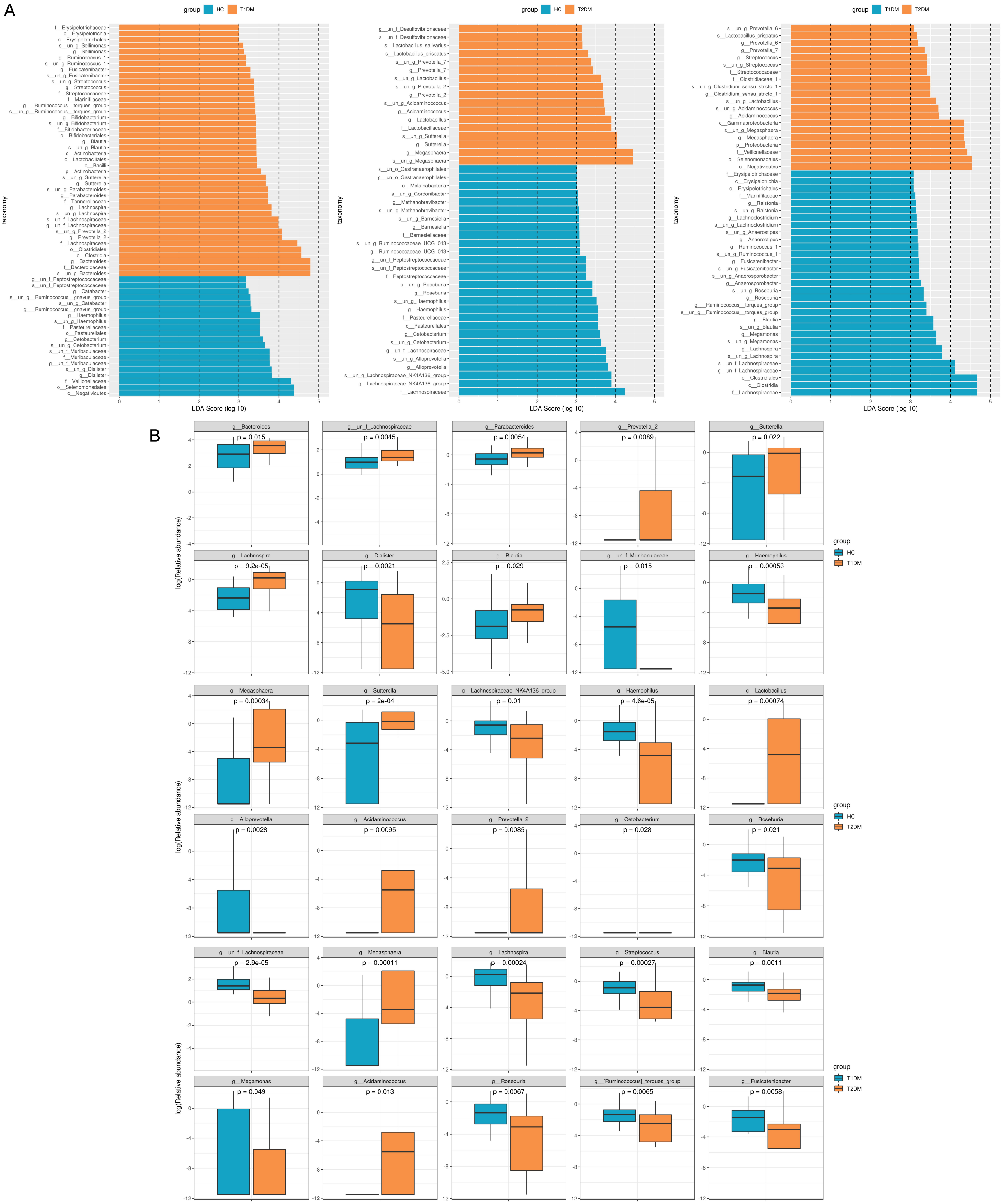
A
B
LDA Score (log 10)
taxonomy
log(Relative abundance)
group HC T1DM
group HC T2DM
group T1DM T2DM

Figure S6

A

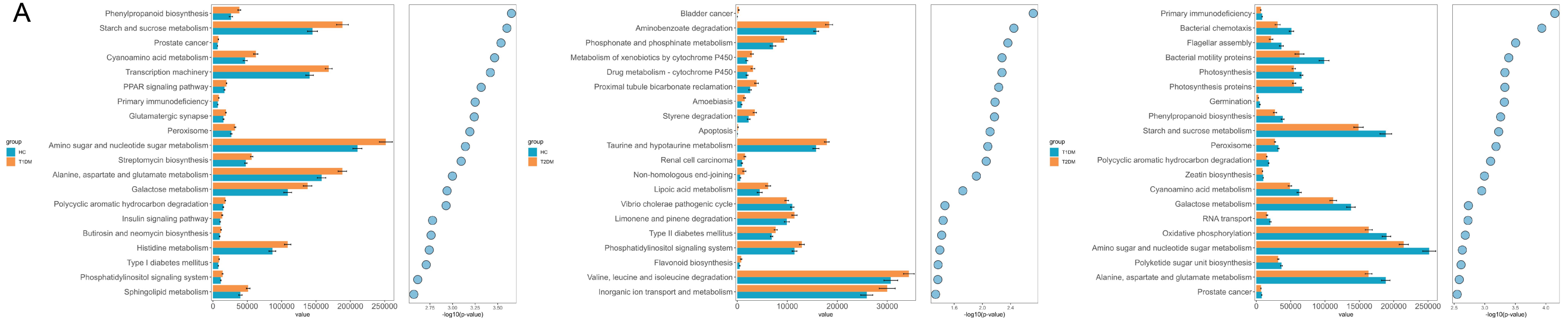

B

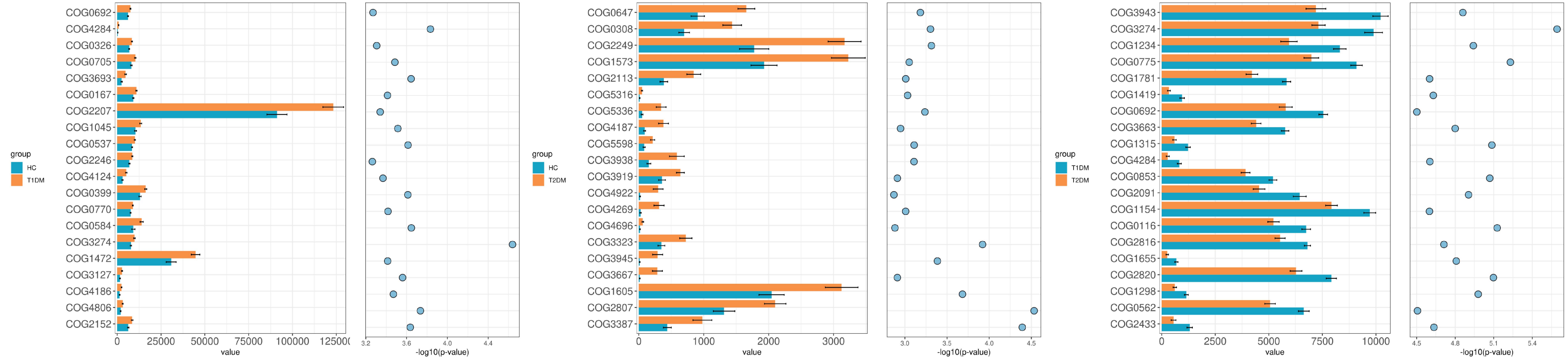

ID	description
COG0692	Uracil DNA glycosylase
COG4284	UDP-glucose pyrophosphorylase
COG0326	Molecular chaperone, HSP90 family
COG0705	Uncharacterized membrane protein (homolog of Drosophila rhomboid)
COG3693	Beta-1,4-xylanase
COG0167	Dihydroorotate dehydrogenase
COG2207	AraC-type DNA-binding domain-containing proteins
COG1045	Serine acetyltransferase
COG0537	Diadenosine tetraphosphate (Ap4A) hydrolase and other HIT family hydrolases
COG2246	Predicted membrane protein
COG4124	Beta-mannanase
COG0399	Predicted pyridoxal phosphate-dependent enzyme apparently involved in regulation of cell wall biogenesis
COG0770	UDP-N-acetylmuramyl pentapeptide synthase
COG0584	Glycerophosphoryl diester phosphodiesterase
COG3274	Uncharacterized protein conserved in bacteria
COG1472	Beta-glucosidase-related glycosidases
COG3127	Predicted ABC-type transport system involved in lysophospholipase L1 biosynthesis, permease component
COG4186	Predicted phosphoesterase or phosphohydrolase
COG4806	L-rhamnose isomerase
COG2152	Predicted glycosylase

ID	description
COG0647	Predicted sugar phosphatases of the HAD superfamily
COG0308	Aminopeptidase N
COG2249	Putative NADPH-quinone reductase (modulator of drug activity B)
COG1573	Uracil-DNA glycosylase
COG2113	ABC-type proline/glycine betaine transport systems, periplasmic components
COG5316	Uncharacterized conserved protein
COG5336	Uncharacterized protein conserved in bacteria
COG4187	Arginine degradation protein (predicted deacylase)
COG5598	Trimethylamine:corrinoid methyltransferase
COG3938	Proline racemase
COG3919	Predicted ATP-grasp enzyme
COG4922	Uncharacterized protein conserved in bacteria
COG4269	Predicted membrane protein
COG4696	Uncharacterized protein conserved in bacteria
COG3323	Uncharacterized protein conserved in bacteria
COG3945	Uncharacterized conserved protein
COG3667	Uncharacterized protein involved in copper resistance
COG1605	Chorismate mutase
COG2807	Cyanate permease
COG3387	Glucoamylase and related glycosyl hydrolases

ID	description
COG3943	Virulence protein
COG3274	Uncharacterized protein conserved in bacteria
COG1234	Metal-dependent hydrolases of the beta-lactamase superfamily III
COG0775	Nucleoside phosphorylase
COG1781	Aspartate carbamoyltransferase, regulatory subunit
COG1419	Flagellar GTP-binding protein
COG0692	Uracil DNA glycosylase
COG3663	G:T/U mismatch-specific DNA glycosylase
COG1315	Predicted polymerase, most proteins contain PALM domain, HD hydrolase domain and Zn-ribbon domain
COG4284	UDP-glucose pyrophosphorylase
COG0853	Aspartate 1-decarboxylase
COG2091	Phosphopantetheinyl transferase
COG1154	Deoxyxylulose-5-phosphate synthase
COG0116	Predicted N6-adenine-specific DNA methylase
COG2816	NTP pyrophosphohydrolases containing a Zn-finger, probably nucleic-acid-binding
COG1655	Uncharacterized protein conserved in bacteria
COG2820	Uridine phosphorylase
COG1298	Flagellar biosynthesis pathway, component FlhA
COG0562	UDP-galactopyranose mutase
COG2433	Uncharacterized conserved protein

Figure S7

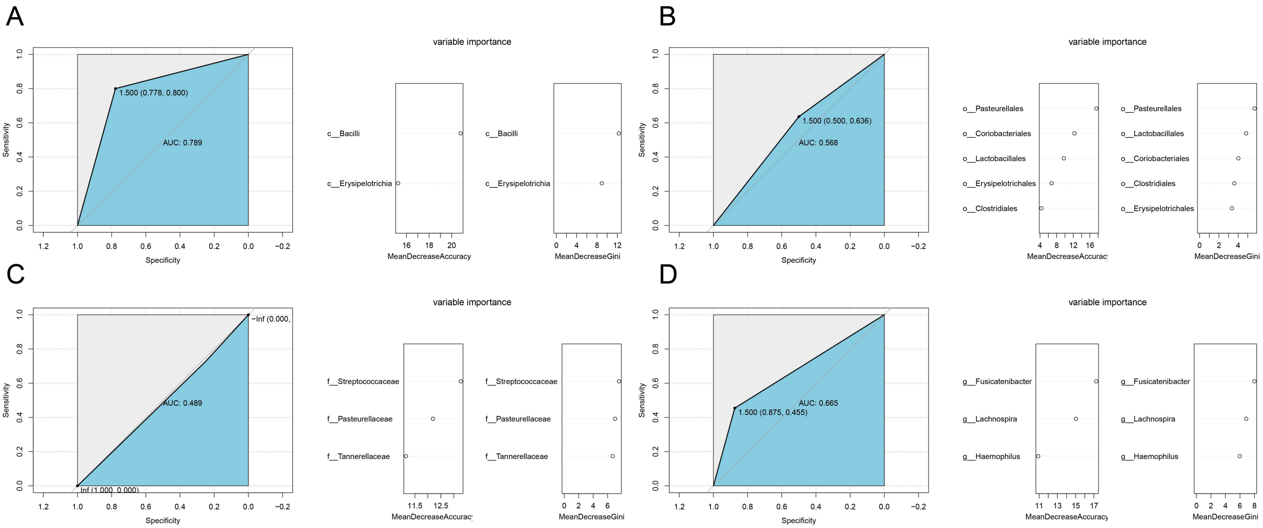

A
B
C
D
variable importance
Sensitivity
Specificity
1.500 (0.778, 0.800)
AUC: 0.789
c__Bacilli
c__Erysipelotrichia
MeanDecreaseAccuracy
MeanDecreaseGini
1.500 (0.500, 0.636)
AUC: 0.568
o__Pasteurellales
o__Coriobacteriales
o__Lactobacillales
o__Erysipelotrichales
o__Clostridiales
−Inf (0.000,
Inf (1.000, 0.000)
AUC: 0.489
f__Streptococcaceae
f__Pasteurellaceae
f__Tannerellaceae
1.500 (0.875, 0.455)
AUC: 0.665
g__Fusicatenibacter
g__Lachnospira
g__Haemophilus

Figure S8

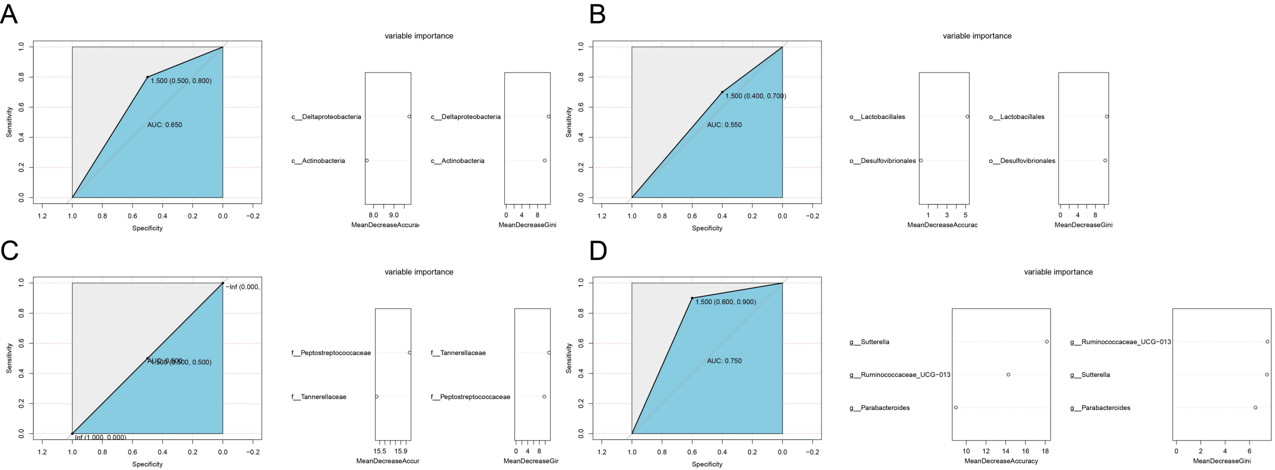

A
B
C
D
variable importance
Sensitivity
Specificity
1.500 (0.500, 0.800)
AUC: 0.650
c__Deltaproteobacteria
c__Actinobacteria
MeanDecreaseAccura
MeanDecreaseGini
1.500 (0.400, 0.700)
AUC: 0.550
o__Lactobacillales
o__Desulfovibrionales
MeanDecreaseAccurac
−Inf (0.000,
Inf (1.000, 0.000)
AUC: 0.500
1.500 (0.500, 0.500)
f__Peptostreptococcaceae
f__Tannerellaceae
MeanDecreaseAccur
MeanDecreaseGir
1.500 (0.600, 0.900)
AUC: 0.750
g__Sutterella
g__Ruminococcaceae_UCG−013
g__Parabacteroides
MeanDecreaseAccuracy
